# Recycling of Stone Sludge for Partial Replacement of Portland Cement and Fine Natural Aggregate in Cement Composites

**DOI:** 10.3390/ma19214477

**Published:** 2026-10-23

**Authors:** Klára Křížová, Rudolf Hela

**Affiliations:** Institute of Technology of Building Materials and Components, Faculty of Civil Engineering, Brno University of Technology, Veveri 331/95, 602 00 Brno, Czech Republic

**Keywords:** stone sludge, waste, by-product, recycling, circular economy, sustainability, mortar, concrete, cement composite, mechanical properties

## Abstract

**Highlights:**

- Replacing up to 15% of the Portland cement with stone sludge does not affect the workability of the mortar.
- Replacing 10% of the Portland cement with stone sludge does not result in a sig-nificant deterioration in the mechanical properties of the mortar.
- Waste stone sludge is a promising alternative for replacing up to 20% of natural fine aggregate.

**Abstract:**

The study examines the use of stone sludge generated during the processing of natural stone. The aim is to verify its benefits in the production of concrete at stonemasonry workshops, particularly with regard to reducing the volume of by-products produced and the consumption of natural raw materials. The experiment focuses on analysing the material parameters of selected types of stone sludge, and on the production of mortars and concretes with varying proportions of sludge. The mortars were evaluated using stone sludge as a partial replacement for cement at 5, 10 and 15%, and for fine natural aggregate at 20, 35 and 50%. The study also aims to assess the effect of further processing by grinding stone sludge and the subsequent impact on the workability of fresh mortars and the resulting flexural and compressive strength. The next stage involved verifying the key properties of the concretes and assessing the potential for reducing the use of natural raw materials and cement whilst still achieving the required mechanical properties. The results obtained evaluate the potential for using stone sludge in the construction industry, particularly its impact on the properties of cement composites, the conservation of natural resources and the reduction in waste sent to landfill.

## 1. Introduction

The construction sector plays an irreplaceable role in the processes of social development and urbanisation [1]. These processes consume vast quantities of raw materials, which in turn leads to a depletion of natural resources and, at the same time, an increase in environmental pollution [2,3]. Construction generates a large volume of construction waste, which is considered the single largest contributor to landfill waste worldwide. On a global scale, construction waste accounts for 16–60% of landfill waste [4]. The most effective strategy for avoiding the disposal of construction waste in landfills is a strategy of waste management and recycling, which is more environmentally beneficial than simple disposal in landfills [4,5]. When reducing the environmental impact of waste, care is taken to adhere to the key ‘3R’ strategies: Reduce, Reuse, Recycle [6].

Waste sludge is a by-product of various types of industrial plants and wastewater treatment works. It consists of both organic and inorganic substances, which can be treated and adapted according to their intended use. The most common methods of sludge treatment include drying, lime stabilisation, thermal treatment and composting [7]. Types of waste sludge are classified according to their origin, which is key to their subsequent utilisation. Each individual sludge has its own unique composition and properties [8]. Kamel Al Zboon et al. [9] investigated the potential for using waste sludge from stone cutting in the production of terrazzo paving, with the aim of minimising landfill disposal whilst taking environmental considerations into account. In the study, sludge was added to a standard mixture in various proportions, and it was found that the strength of the paving tiles was not reduced when up to 25% of the mixture consisted of waste sludge. Zhan Bao Jian et al. [10] focused in their study on the conversion of sludge from textile wastewater into a construction material for civil engineering works. For example, in the production of concrete blocks, pre-treated textile sludge was used, from which ammonia had been removed using a lime-based material. The research findings indicate that, for the production of non-load-bearing concrete blocks, it is possible to replace fine natural aggregate with textile sludge up to a maximum of 20%. Another sophisticated solution involves converting stone sludge into artificial stone slabs. Barani and Esmaili [11] conducted a study on the production of artificial stone slabs comprising approximately 50% marble and granite sludge, 12% ground quartz, 25% waste glass and 13% resin, using vibratory compaction in a vacuum environment. The resulting slabs possess excellent physical and mechanical properties and an attractive appearance, and are suitable for use as wall cladding, floor tiles, windowsills or kitchen worktops.

The ornamental and dimension stone processing industry is one of the least environmentally friendly sectors, producing large quantities of waste [12]. Waste generated by rock processing is the most common form of waste produced during all stages of stone processing [13]. The ever-increasing accumulation of this type of waste in landfills causes serious environmental pollution, particularly when the sludge begins to dry out. It then turns into fine dust, which can be blown by the wind and cause air pollution in the surrounding area. This air containing fine dust can cause serious respiratory diseases, skin problems and eye conditions among local residents and animals. Waste sludge from metamorphic, igneous or sedimentary rocks is produced as a by-product in the ornamental stone industry during various stages of stone processing, such as cutting, shaping or various surface treatments, for example stone polishing [14,15,16].

Currently, the biggest trend is to incorporate by-products from the construction, energy and metallurgical industries into the production processes of concrete and mortar as suitable substitutes for fine aggregate or cement, up to a certain optimal level [17,18]. The use of waste products, in the form of stone-cutting sludge, also entails significant drawbacks. Adding large quantities of these fine particles can result in high water consumption, which carries risks associated with volume changes and a reduction in physical and mechanical properties [19,20]. The shape, specific surface area and structure of fine particles have a fundamental influence on the properties of fresh concrete/mortar, particularly on workability and the amount of mixing water [21]. Siddique et al. [22] studied the workability of fresh concrete with the addition of marble sludge (range 0–20%) as a cement substitute. The ground marble powder contained in the sludge had a particle size range of 0–90 µm and consisted of 99% CaCO_3_. All the mixtures tested had the same water/cement ratio of 0.42. The workability of fresh concrete was measured using the Slump test, in which the reference mix (without marble powder) had a slump value of 88 mm, whilst the mix with the highest marble powder content (20% by weight) had a value of 74 mm, representing a 15% reduction in workability. The authors attribute this deterioration to the influence of grain shape. In contrast to these results, studies by other authors can be found in which the same or better workability of the fresh mix was achieved. For example, Munir et al. [23] investigated concrete with up to 40% of the cement replaced by this material. Compared to previous studies, the powder was ground in a ball mill to improve the grain shape and break up agglomerates in the marble sludge. The ground powder achieved a specific surface area almost identical (269 m^2^/kg) to that of the cement used. The results of the experiment on the mixture with 40% substitution demonstrated a 5% improvement in workability. It is evident from the results that substituting cement up to 20% has no significant effect on the development of compressive strength. This is due to the filling of micropores and the compaction of the matrix. A higher rate of cement substitution, however, has a negative impact on strength [14].

The study of Gupta and Vyas [24] focused on incorporating sludge into mortars as a substitute for fine aggregate. The study’s results indicate that mortar with 40% of the sand replaced by stone sludge provides the highest compressive strength compared with the reference sample. Mortar prepared with 30% sludge content exhibited comparable shrinkage values to the reference mortar. Conversely, at higher replacement rates, shrinkage increased rapidly. Corinaldesi et al. [25] investigated marble sludge in mortar production in their study. The research confirmed that a high fineness of marble sludge (above 1500 m^2^/kg) can improve the cohesion of the mixture. With regard to mechanical properties, it was found that replacing 10% of the sand with marble sludge, in the presence of a superplasticizer, yielded maximum compressive strength whilst maintaining the same workability. Furthermore, in all the mixtures tested, the marble sludge had a positive effect on the mechanical properties during the initial stage of hydration. The results of the experiment by Mishra et al. [26], in which sludge produced from marble grinding was used, showed that increasing the replacement of natural aggregate led to an improvement in strength characteristics, without any changes to the microstructure of the concrete composite. Another study by Soliman [27] investigates the effect of marble sludge on precast reinforced concrete elements. The results showed that using marble sludge as a cement replacement at a dosage of around 5% improves workability, resulting in increased compaction and a subsequent improvement in compressive strength and flexural strength. In his study focusing on the production of green concrete through the addition of granite sludge, Allam [12] states that replacing 10% of the sand with granite sludge ensures higher compressive strength at all ages of the concrete under investigation. When cement was replaced with sludge, none of the mixes resulted in an increase in strength. 

The study is designed as a combinatorial study involving a systematic comparison of three different types of stone sludge (marble, sandstone, and mixed), two states of use (raw and ground), and two methods of substitution (partial replacement of Portland cement or fine natural aggregate). The study’s contribution lies in its experimental verification demonstrating the compatibility of individual substitutions in various proportions, taking into account the origin of the stone sludge in mortars and concrete. 

## 2. Materials and Methods

The current trend is towards eco-conscious thinking and an environmental perspective, which is evident across all sectors of the construction industry. This study aims to assess the technical aspects of using waste stone sludge as a partial substitute for cement, fine natural aggregate of the 0/4 mm fraction, or a combination of the two, in the production of cement composites. The study focuses on determining the basic material characteristics of suitably selected types of stone sludge and evaluating the potential for their further use in the production of mortars and concretes.

### 2.1. Raw Materials

Portland cement CEM I 42.5 R according to EN 197-1 [28] was used as binder. 

The aggregate used was standard silica sand, which is used for the production of standard mortars according to EN 196-1 [29]. Natural aggregate was used in the 0/4, 4/8 and 8/16 mm sizes. The coarse aggregate 4/8 mm fraction consisted of quarried aggregate, whilst the 8/16 mm fraction consisted of crushed aggregate. 

Drinking water from the public water supply system was used to produce the experimental samples.

A polycarboxylate-based superplasticizer from the Sika Group (Brno, Czech Republic) was used to regulate the amount of mixing water. 

**Figure 1:**
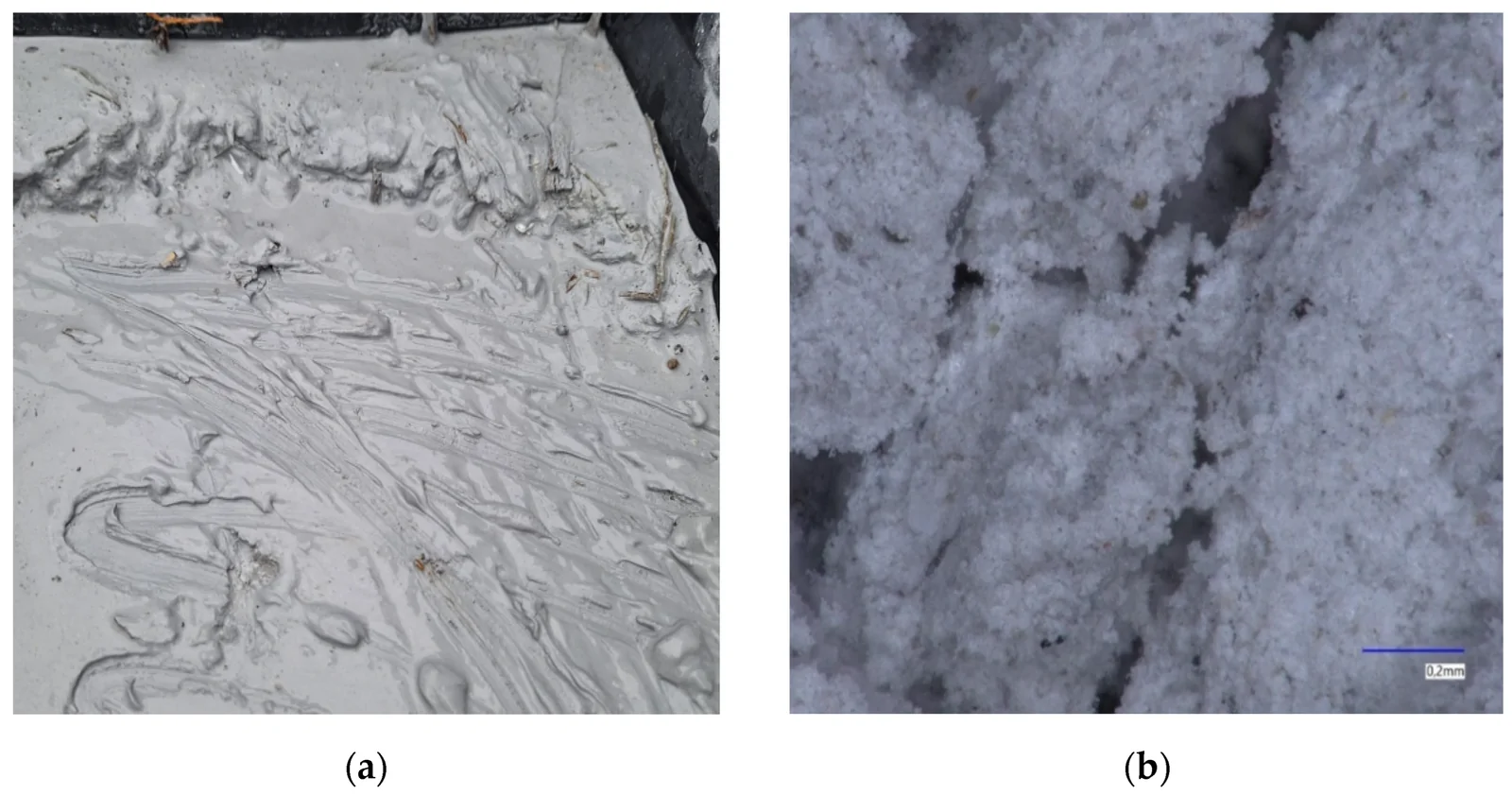
A sample of marble sludge (**a**) and a microscope image (**b**).

**Figure 2:**
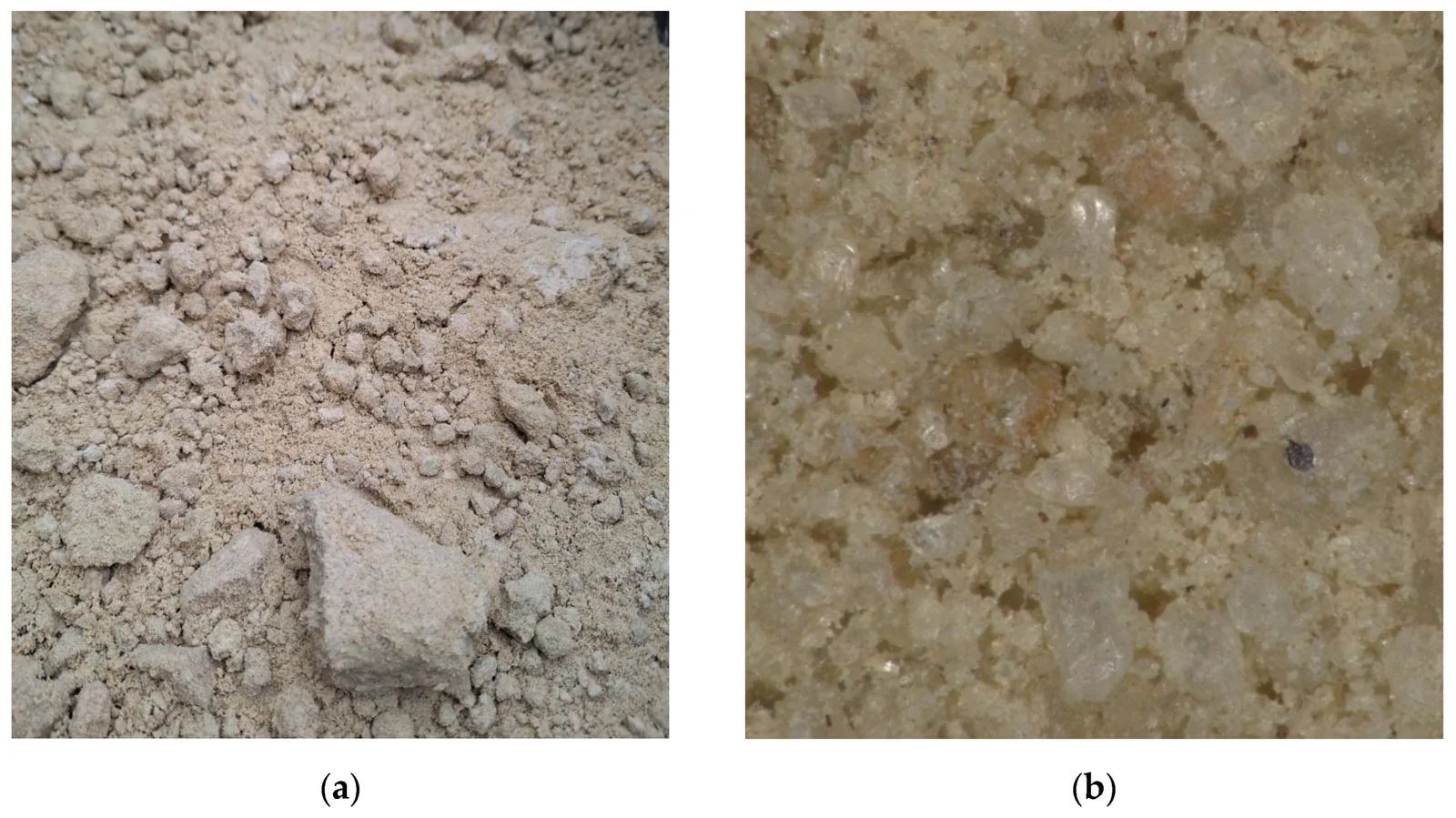
A sample of sandstone sludge (**a**) and a microscope image (**b**).

**Figure 3:**
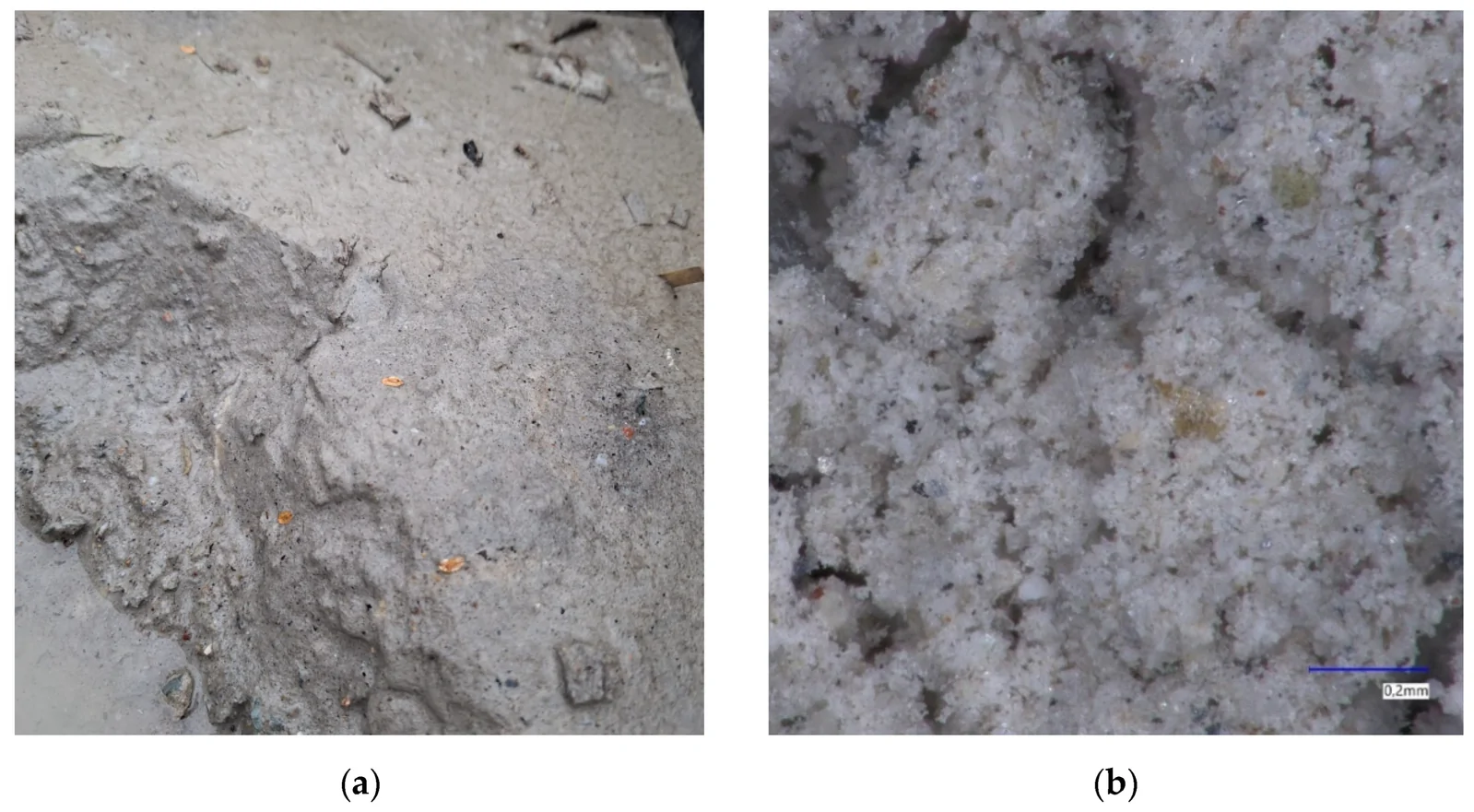
A sample of mixed sludge (**a**) and a microscope image (**b**).

Stone sludge is a by-product of cutting and grinding the original rock. In the experiment, sludge is used as a substitute for cement and fine aggregate. To a limited extent, it is possible to specify stone sludge in accordance with EN 12620. This involves determining the grain size/fraction size and bulk density. It would also be possible to consider setting grain size requirements for filler as aggregate in accordance with EN 933-10 (Table 7 in the standard). The basic description and details of the stone sludge are given in Table 1 and Figure 1, Figure 2 and Figure 3. Some of the dried sludge samples were processed by grinding in a Reutsch RS 200 vibrating disc mill for 3 min at a speed of 1000 revolutions per minute. The reason for grinding the dried slurries was to increase their specific surface area, which should ensure higher activity and better utilisation in the cement composite, thereby ensuring greater reactivity of the sludge during hydration. A positive effect was expected, particularly on the mechanical properties of the resulting composites, through the disintegration of agglomerates and modification of the particle size distribution.Figure 1 shows marble sludge (abbreviation) produced mainly during the cutting of the original rock. VYDRUS spol. s.r.o. (Králíky, Czech Republic) specialises in marble processing and the manufacture of paving stones.

Figure 2 shows sandstone sludge (abbreviation) produced mainly during the cutting of sandstone. TEKAM ZAMEL s.r.o. (Záměl, Eastern Bohemia, Czech Republic) specialises in the stoning and all aspects of sandstone processing. 

Figure 3 shows mixed sludge (abbreviation) produced by cutting, grinding and polishing various types of stone. The sludge was supplied by Stone carving Holanec (Opočno, Czech Republic), which uses a variety of domestic and foreign materials in its stonemasonry production. In addition to traditional materials such as granite, marble and sandstone, the company also specialises in working with artificial stone made from terrazzo.

The specific gravity of dried (raw/ground) sludge and CEM I 42.5 R cement was determined in accordance with the CSN 72 2113 standard [30]. This parameter can be defined as the mass of 1 m^3^ of cement (sludge) excluding voids and pores, expressed in kg/m^3^. This test was carried out using a Micromeritics AccuPyc II 1340 automatic helium pycnometer (Malvern Panalytical, Malvern, Worcestershire, UK).

The fineness of grinding is defined by the specific surface area, which was measured using an automatic PC–Blaine–star instrument (Toni Technik Baustoffprüfsysteme GmbH, Berlin, Germany) in accordance with standard EN 196-6 [31]. The instrument operates on the principle of the flow method. The result is the average of two specific surface area values in m^2^/kg. 

**Figure 4:**
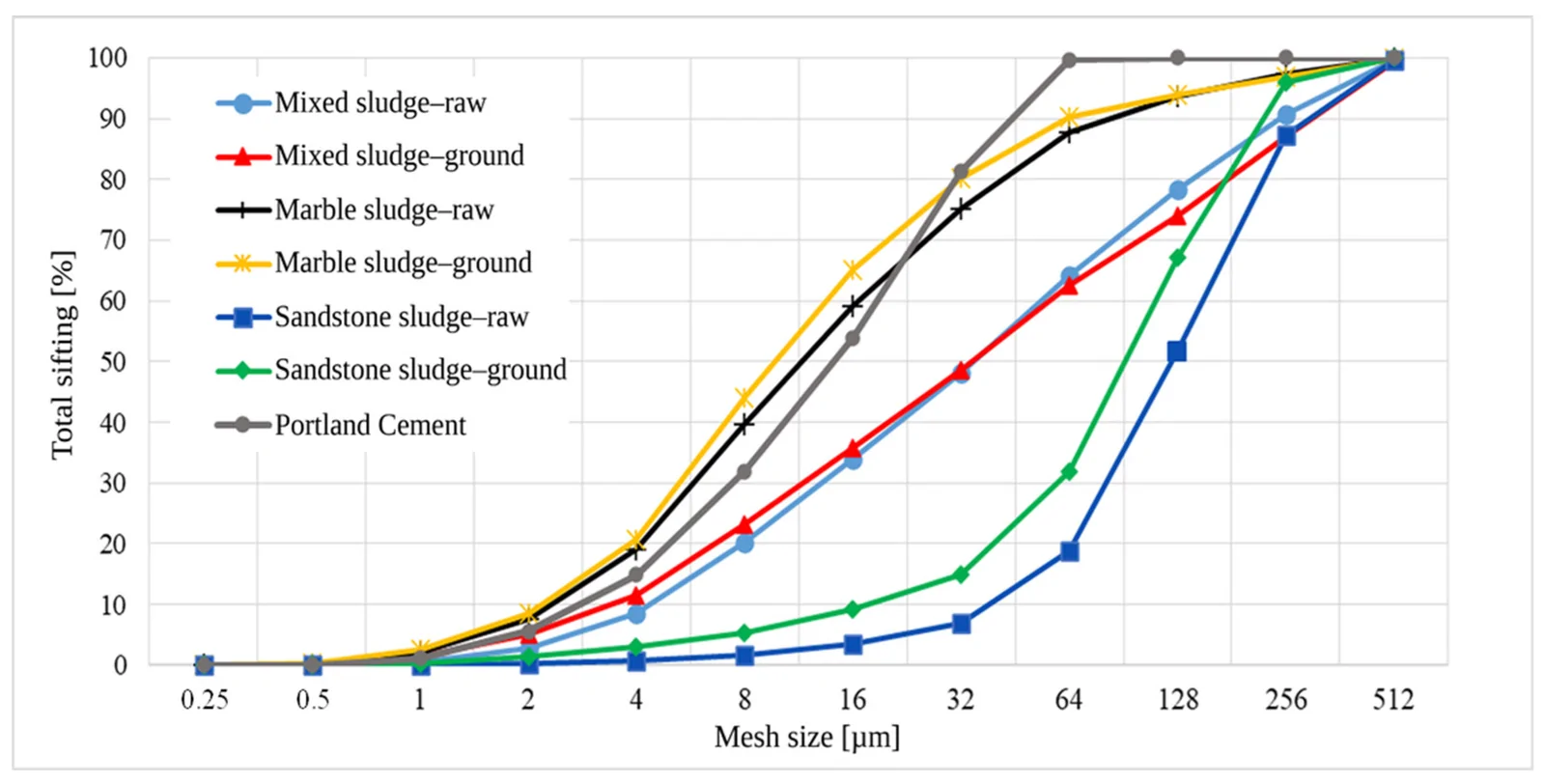
Particle size distribution for sludge (raw/ground) and Portland cement.

Particle size distribution was determined using a Mastersizer 2000 laser particle size analyser (Malvern Panalytical, Malvern, Worcestershire, UK) in accordance with EN ISO 13 320 [32] (Figure 4). The instrument is designed to assess the particle size distribution of materials up to a particle size of 2 mm. The instrument determines particle size distribution and sieve analysis. Moisture measurement using the gravimetric method falls within the group of direct methods. This is a highly accurate method for determining the moisture content of a sample, whereby the sample is dried and the moisture content is determined based on the loss in mass during drying. Drying of the sample ceases when the mass of the test sample no longer changes as a result of drying. The measurement results are expressed as mass percentages. The measurements were carried out in accordance with EN 1097–5 [33]. The results of the analyses are shown in Table 1. Table 2 sets out the basic information superplasticizer.

**Figure 5:**
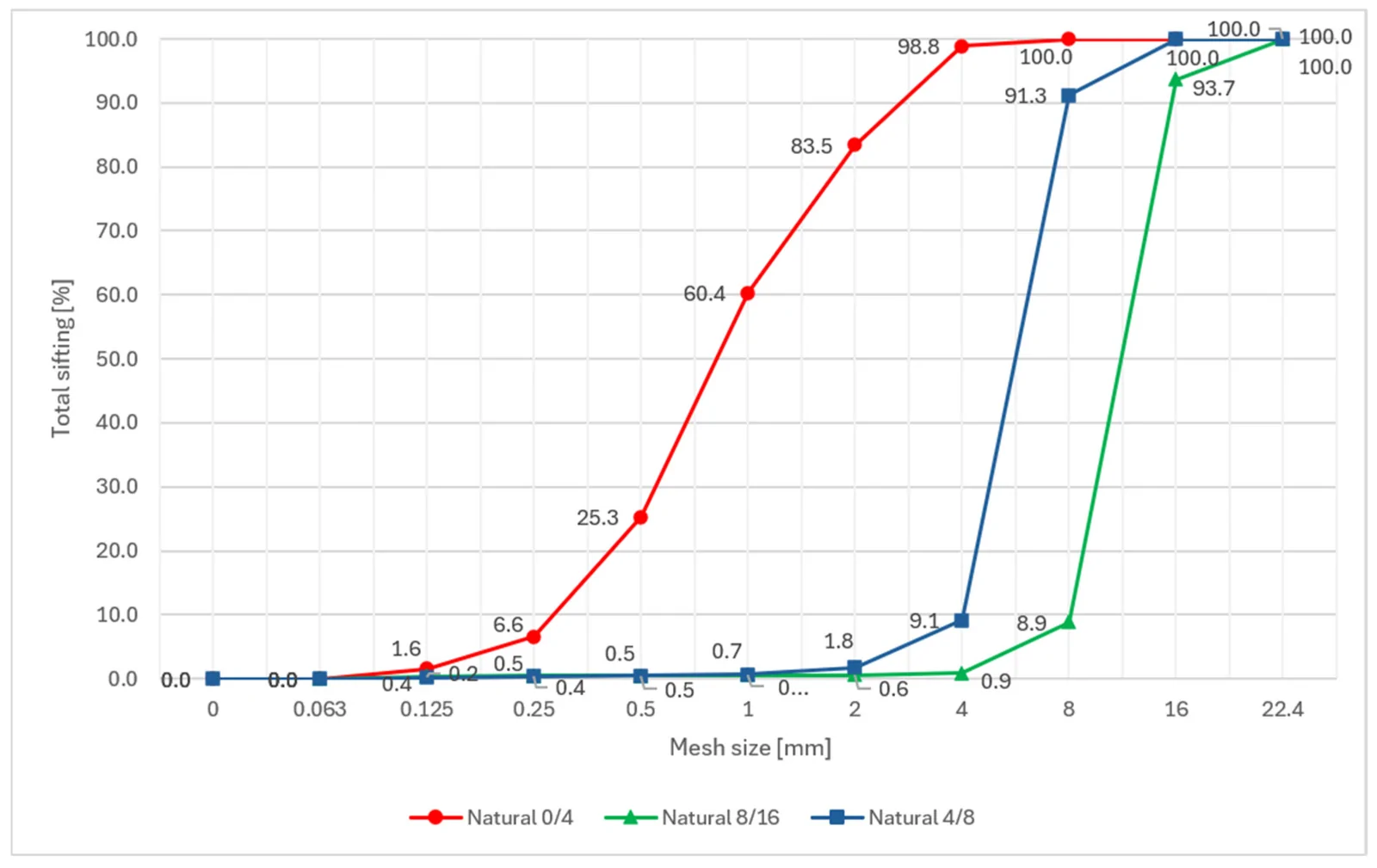
Particle size distribution for natural aggregates in the 0/4, 4/8 and 8/16 mm fractions.

The particle size distribution of natural aggregate in the 0/4, 4/8 and 8/16 mm fractions was determined in accordance with EN 933-1 [34] and is shown in Figure 5. First, representative samples were prepared by quartering, and the test samples were then dried to constant weight at 110 °C. The percentage content of the individual fractions in the aggregate samples was determined using a standard set of sieves. All aggregate used in concrete and aggregate in mortars, where the fine fraction of aggregate was being substituted, was subjected to this test.

### 2.2. Mix Designs and Experimental Methods of Mortar and Concrete

In the first stage, standard mortar was produced in accordance with EN 196-1 [29]. The mortar was produced using Portland cement CEM I 42.5 R, standardised sand (CEN EN 196-1) and stone sludge. First, a reference mortar was produced, followed by mortars with 5, 10 and 15% of the cement replaced by dried stone sludge. A constant proportion of sand and water was used in all formulations. Six test specimens measuring 40 × 40 × 160 mm were produced from each mixture. The formulations given below (Table 3) were used for both stone sludge treated by drying alone and sludge that had been both dried and ground. All mortars were mixed to achieve the same consistency.

In the second stage, cement mortars were produced in which the 0/4 mm fraction of natural aggregate was replaced with stone sludge. In this part of the study, a reference mortar was first produced, followed by mortars containing 20, 35 and 50% stone sludge in place of natural fine aggregate. For all formulations (Table 4), the aim was to achieve the same consistency to facilitate comparison of the results. For this reason, it was necessary to increase the amount of mixing water as the aggregate replacement increased. Six test specimens measuring 40 × 40 × 160 mm were produced. The test specimens were stored in water environment conditions (in accordance with EN 196-1, 8.3) and were used to determine compressive strength and flexural tensile strength after 7 and 28 days of curing. In view of the results for the mortars from the first part of this stage, it was decided that, in this part, only mortars containing dried stone sludge that had not been further processed by grinding would be produced.

The consistency tests on the shaking table were carried out in accordance with the procedures set out in EN 1015–3 [35]. The bulk density of hardened cement mortars (specimens 40 × 40 × 160 mm) was determined in accordance with EN 1015–10 [36]. The bulk density in the fresh state was calculated as the ratio of the mass of the fresh mortar to the volume of the corresponding mould and rounded to the nearest 10 kg/m^3^. In accordance with EN 196-1 [29], test specimens measuring 40 × 40 × 160 mm were first subjected to a flexural tensile strength test and then to a compressive strength test. Both of these tests were carried out after 7 and 28 days of curing [37]. The final test carried out on the manufactured mortars was the determination of the efficiency index in accordance with EN 450-1 [38].

Based on an evaluation of the results obtained from the mortars (Stages I and II), a series of concrete samples was produced (Stage III). The aim of the third stage was to verify the potential of the concretes (REC 1 and REC 2), which reduce the quantity of fine natural aggregate and cement, to effectively reduce the amount of waste that would normally be sent to landfill. Both modified mixes, REC 1 and REC 2 (Table 5), reduce the amount of cement used by 10%; at the same time, REC 1 reduces the amount of 0/4 mm fine natural aggregate used by 20%, whilst REC 2 reduces it by 35%. The substitution of aggregate and cement with stone sludge was carried out by directly replacing the removed portion, measured in kilograms, with sludge. Subsequently, the composition of the mixes was recalculated according to the moisture content of the individual stone sludge. The water content of the individual sludge was added to the mixing water; for this reason, the stone sludge in the mix table represents only its dry component.

The concrete specimens were produced in accordance with EN 206+A2 [39] to the same consistency class. Test cubes measuring 150 × 150 × 150 mm and beams measuring 100 × 100 × 400 mm were produced to determine the bulk densities in the fresh and hardened states, and subsequently to determine the compressive and flexural strengths after 7 and 28 days of curing, as well as resistance to water penetration under pressure. The slump test was carried out in accordance with standard EN 12350-2 [40]. The test to determine the bulk density of fresh concrete was carried out in accordance with standard EN 12350-6 [41]. The method is based on knowledge of the volume of the test container, its filling, compaction and subsequent weighing. The bulk density of fresh concrete is rounded to the nearest 10 kg/m^3^.

The bulk density of hardened concrete was determined in accordance with EN 12390-7 [42]. The test specimens were weighed to an accuracy of 0.01 kg and their dimensions were measured. The resulting value was expressed as the average of the measurements, rounded to the nearest 10 kg/m^3^. Compressive strength was determined in accordance with the procedure set out in EN 12390-3 [43]. This parameter expresses the relationship between the external force acting on the test specimen and its loaded area up to the point of failure. A further mechanical test involved determining tensile strength by three-point bending in accordance with EN 12390-5 [44]. The test involves subjecting the test specimens to a bending moment resulting from the load transmitted via the upper and lower supports. The depth of penetration of water under pressure method was determined in accordance with EN 12390-8 [45]. The measurement is carried out by applying a water pressure of 500 kPa for a period of 72 h. After 72 h, the specimen is broken and the maximum depth of water penetration is recorded to the nearest millimetre.

## 3. Results

### 3.1. Determination of Fresh Properties of Mortar

The results for mortars produced during Stage I, in which cement was replaced by dried stone sludge (5, 10 and 15%), show that the selected cement substitutes had no adverse effect on the consistency of the fresh mortar. The same hypothesis is presented in study [23], which gives a positive assessment of substitutions of up to 20%. The bulk density values remained essentially unchanged, even when ground sludge was used. The results do not deviate significantly from those of the reference mortar. Table 6 below summarises the results obtained.

Table 7 shows the consistency and bulk density values determined for fresh mortars when fine aggregate was replaced with stone sludge (Stage II). This sludge was not further processed by grinding, but only by drying. The results obtained demonstrate a significant effect of the substitutions on the consistency of the fresh mortar. To maintain the same consistency, it was necessary to increase the amount of mixing water in all formulations where fine aggregate was substituted. Mortars with a 20% substitution still performed very well in the fresh state; with higher substitutions, stickiness gradually increased. To achieve the same workability, mortars containing sludge required more mixing water. Mortars with a 20% substitution required approximately 15% more mixing water to achieve the same spread value; otherwise, they did not exhibit any deterioration in handling behaviour. For mortars in which 50% of the fine aggregate was replaced, the water content had to be increased by up to 60%. In terms of workability, the mortar with 20% replacement proved to be the best. A downward trend in bulk density values in the fresh state can be observed as the proportion of waste sludge increases.

### 3.2. Determination of Hardened Properties of Mortar

The bulk density in the hardened state was calculated as the ratio of the mass of the test specimens to their volume, which was calculated from their actual dimensions. The flexural tensile and compressive strength of the mortars was carried out on test specimens with dimensions of 40 × 40 × 160 mm. The strength test is based on the principle of destructive failure of the test sample. Compressive strength was performed on fractions of the specimens after the flexural tensile strength test. A prescribed pressure is applied to the specimen over a test area of 40 × 40 mm and the maximum failure force is recorded.

The reactivity of stone sludge was determined according to the reactivity index specified in EN 450-1 [38]. The reactivity index is typically determined on compressed cement mortars (specimens 40 × 40 × 160 mm, produced in accordance with EN 196-1 [29]) as the ratio of the compressive strength of the mortar containing the additive (25%) to that of the mortar without the additive. Fly ash complies with the standard if its compressive strength after 28 days reaches at least 75% of the compressive strength of the reference mortar. The determination of the efficiency index for stone sludge was inspired by the aforementioned procedure, as no test method is specified for these waste products. In this study, the efficiency index was monitored at a 15% cement replacement rate, using both ground and unground sludge.

#### 3.2.1. Replacing Portland Cement with Waste Material

Table 8 and Table 9 show the results for bulk density and compressive and flexural tensile strengths obtained for mortars at 7 and 28 days of age (Stage I). For most results, there was a trend towards a decrease in bulk density over time, which is a well-known fact: as water evaporates, the mass of mortars or concrete decreases. The greatest differences in bulk density were observed in the results for sandstone sludge, which had the lowest specific surface area. The hypothesis that a lower bulk density results from the use of sludge characterised by a lower specific surface area compared with cement is also confirmed by the research of Raswan et al. [46]. In the results for mortars where the ground variant was used, these values increased, demonstrating the influence of particle size. In other cases, there was a slight reduction in values compared with the reference mortar.

The benefit of grinding the raw materials was demonstrated only in the case of sandstone sludge; the other samples generally achieved similar values to those obtained when using raw sludge. Mortars containing stone sludge did not show such significant differences in strength compared with the reference mortar when comparing the values from the 7th and 28th days. Of particular interest is the increase in flexural tensile strength in mortars with 5% and 10% marble sludge, 5% sandstone sludge and 5% mixed sludge, which is likely due to the incorporation of more inert particles, leading to lower volume changes. The results of the destructive tests were used to identify promising substitutes that could be used in concrete in the future. Replacements of 5% and 10% appear to be promising due to their higher, similar or slightly lower strength characteristics when compared with the reference mix design. These conclusions are consistent with the experimental findings of study [14], which states that replacements of up to 20% do not adversely affect the development of mechanical properties.

**Figure 6:**
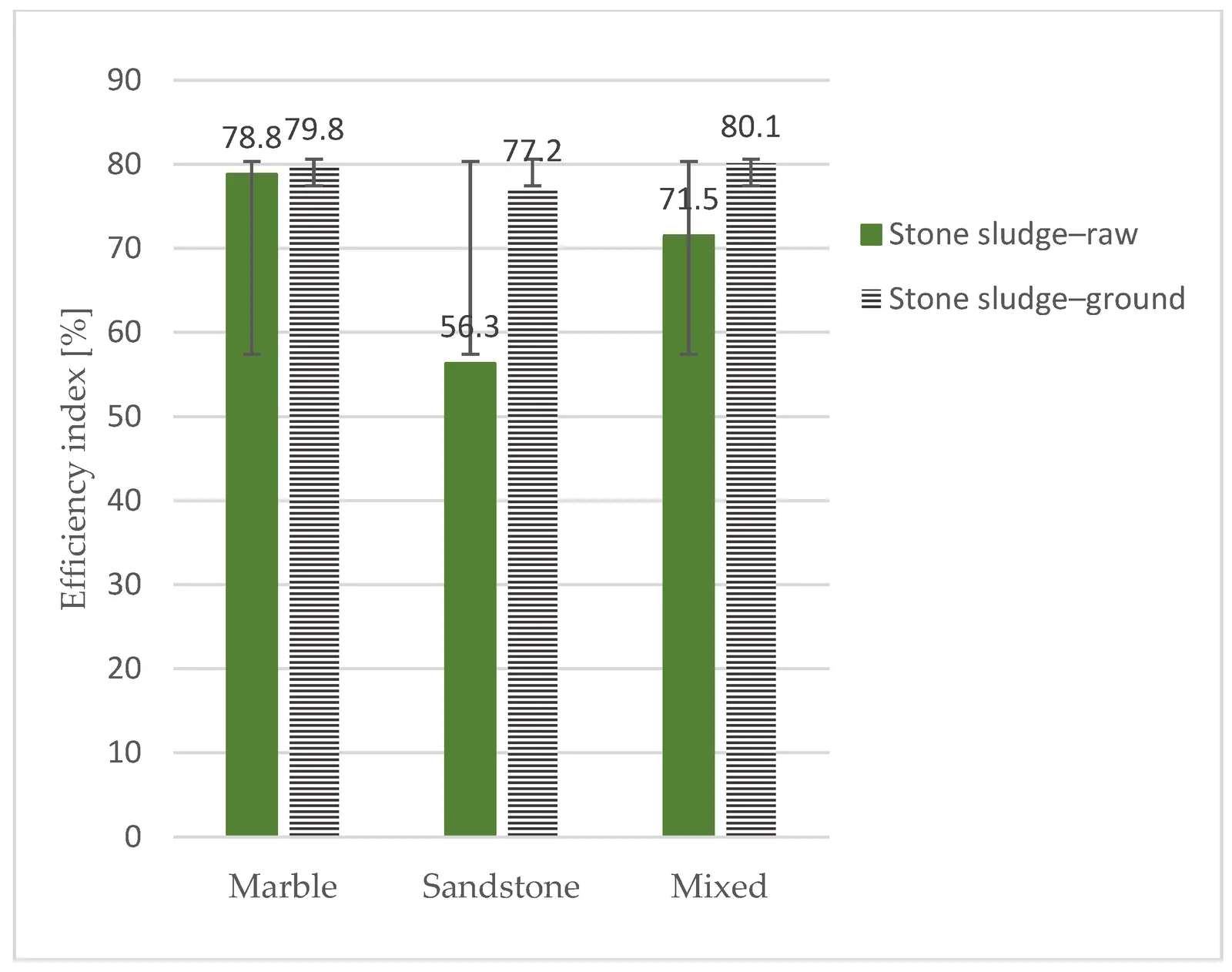
Efficiency index for stone sludge.

The highest efficiency index at 15% cement replacement was achieved by the ground variants of marble and mixed sludge. By contrast, the unground sandstone sludge achieved the significantly lowest values after 28 days. In Figure 6, it can be seen that grinding had almost no effect on the activity index of the marble sludge. On the other hand, the activity index for sandstone sludge increased considerably. The results for mixed sludge also improved with grinding, but not significantly.

#### 3.2.2. Replacing Fine Natural Aggregate with Stone Sludge

The bulk density and strength obtained reflected the amount of water that had to be added to the mortars to achieve the same workability. For these reasons, the mechanical properties deteriorate as the replacement of fine aggregate increases (Table 10). The bulk density in the hardened state followed a similar trend to those in the fresh state, in that they gradually decreased as the percentage replacement of the fine aggregate fraction increased. The use of stone sludge led to the expected reduction in mechanical properties for all mixes at both 7 and 28 days of age. Data presented in [2] indicate that the reduction in compressive strength is a consequence of reduced workability. This reduced workability can be attributed to the increased surface area and specific density of the fine particles in the stone waste. The research by Al-Jrajreh et al. [13] reached the similar conclusion. This reduced workability increases the demand for cement paste, resulting in poor compactness, which in turn reduces compressive strength. The mix design with a 20% addition of sandstone sludge achieved a slightly higher compressive strength after 28 days compared with the reference mix design. This increase in strength is attributed to a reduction in the number of microcracks due to the lower cement content in the mix design and an increase in matrix density due to the fine particles. Conversely, the worst results were achieved by the mix design with 50% of the fine aggregate replaced by mixed stone sludge. In this case, both compressive strength and flexural tensile strength were reduced by approximately 40% after 28 days compared with the reference mix design. Studies [2,12,25] focusing on assessing the effect of marble sludge conclude that a 10% replacement is optimal for achieving strengths comparable to those of the reference mortar. The results of this study indicate that the optimum replacement rate is 20% for fine natural aggregate of the 0/4 mm fraction.

### 3.3. Determination of Fresh and Hardened Properties of Concrete

**Figure 7:**
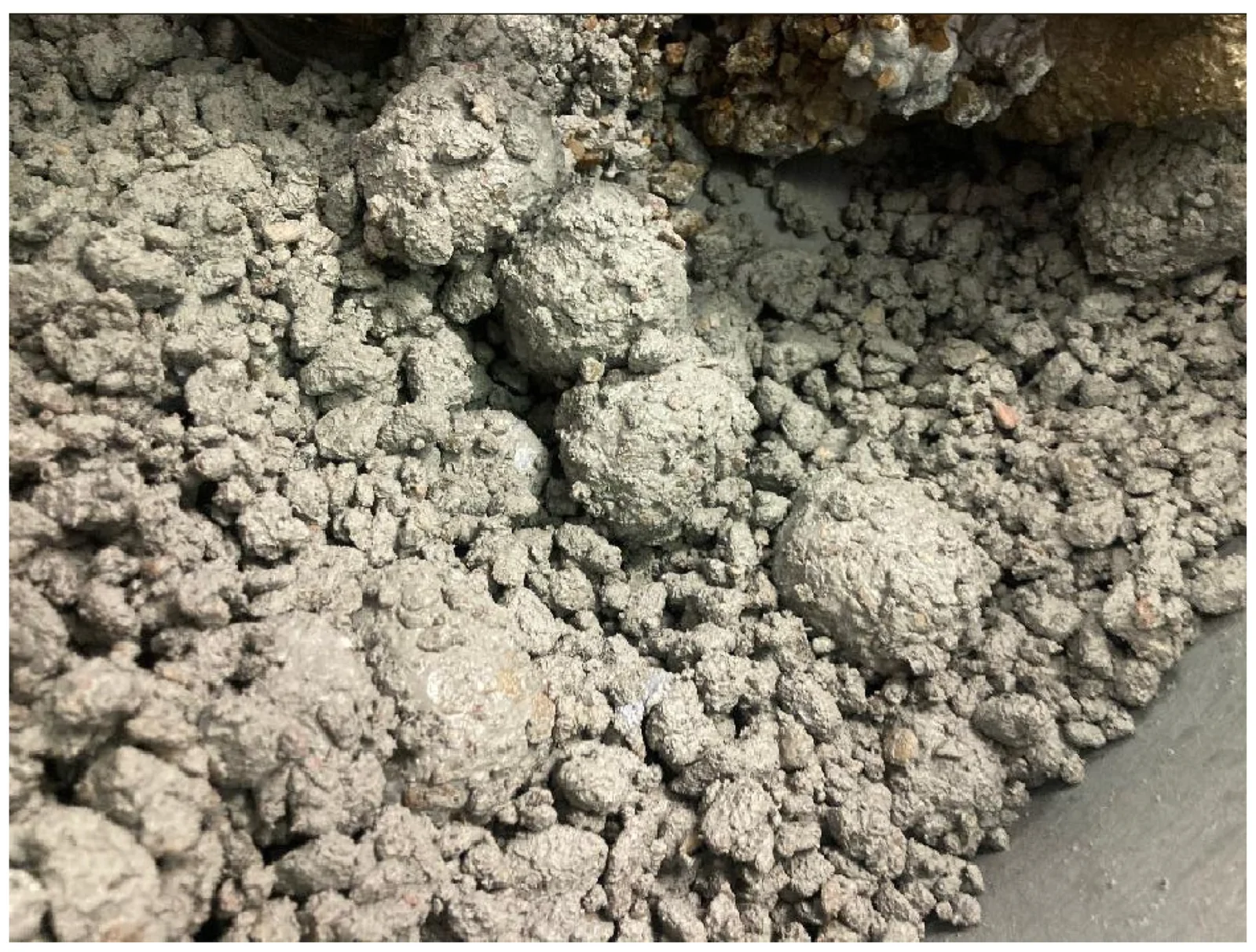
Clumps of sludge during the concrete mixing process.

**Figure 8:**
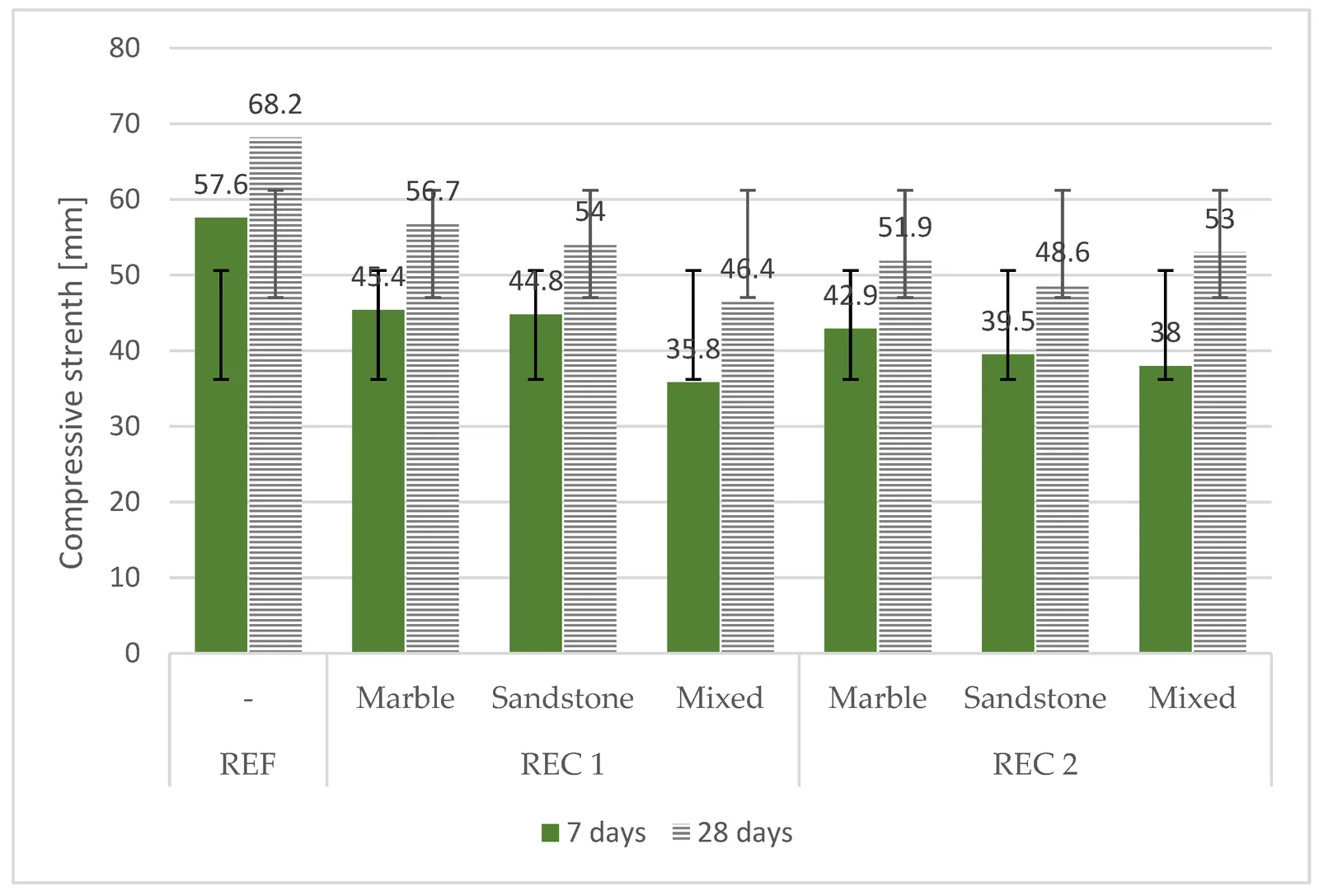
Strength development over time for reference concrete and concrete containing stone sludge.

The results and their evaluation are based on a combination of two different substitutions (10% Portland cement and 20 or 30% fine natural aggregate) in the proposed mixtures. The concrete in Stage III was mixed to a uniform consistency (S4). The quantity of superplasticiser was fixed at 0.8% of the cement content for concretes including stone sludge (REC 1, REC 2) and 1.0% for reference concrete (REF). A problem encountered during mixing was the formation of sludge clumps (Figure 7), which was successfully eliminated by modifying the mixing procedure. The values shown in Table 10 and Figure 8 are always the average of three test specimens from each mix design, depending on the type of waste sludge used.

The results for the density of hardened concrete containing sludge (REC 1 and REC 2) showed a reduction in density of up to 5% compared with the reference concrete (REF) (Table 11). 

The compressive strength results confirmed that the addition of stone sludge has no positive effect on compressive strength (Table 10). This finding is also supported by the research of Kumawat et al. [15], who state that the reduction occurs as a result of the higher silt content in certain types of sludge and higher water absorption, thereby reducing the density of the concrete. However, in the case of concrete containing sludge, the same trend in strength development was observed as in the reference concrete (REF) containing only Portland cement and fine natural aggregate 0/4. Furthermore, the trend in strength development remains the same even when the fine natural aggregate 0/4 content is reduced by 20% (REC 1) or 35% (REC 2). The increase in strength in concrete containing stone sludge ranged from 15–20% after 7 and 28 days. Figure 8 shows the compressive strength results achieved for the individual concrete specimens. Concrete specimens containing marble sludge exhibited the lowest reduction in compressive strength, at approximately 20%. The worst result was achieved by the REC 1 mixed sludge, where strength was reduced by approximately 35%.

In all cases where stone sludge was used, the flexural tensile strength showed a downward trend. The highest values were recorded for sandstone sludge, which achieved values approximately 15% lower than the reference mix. In terms of the results achieved, the REC 1 mixed sludge performed the worst. In this case, the reduction in flexural tensile strength was approximately 25%.

The results in Table 12 confirm the importance of the concrete mix design and the influence of the mixing water content. All the concretes produced achieved a depth of penetration of water under pressure of less than 50 mm; they can therefore be classified as watertight in accordance with CSN P 73 2404 (Table F.1.1) [47] for the given exposure environment as defined by EN 206+A2 [39]. The best results were achieved by the reference mix, which contained the smallest amount of mixing water. The other concretes containing stone sludge achieved poorer results. In this test, it was assumed that the large quantity of fine particles contained in the stone sludge would be able to fill the concrete matrix more effectively and reduce the concrete’s water absorption [13]. To adequately confirm durability, it would be necessary to conduct a series of additional tests that take into account the various exposure environments affecting concrete containing sludge as a substitute for primary raw materials.

## 4. Conclusions

This study examined the use of various types of stone sludge and compared their effects on the properties of cement mortars and concrete.

- Based on the results obtained, it can be concluded that replacing up to 15% of the cement does not affect the workability of the mortars. This is likely due to the similarity in the particle size distribution of the stone sludge used and Portland cement. However, the effect of particle size was observed in sandstone sludge, which, in its raw state, exhibited a different particle size distribution owing to the nature of the original rock.
- The results for the compressive strength of mortars show the positive effect of grinding stone sludge. In most cases, mortars containing this sludge achieved an increase in both compressive strength and flexural strength compared with mortars containing raw sludge.
- The effect of grinding stone sludge was also evident in the efficiency index, which was determined for a 15% cement replacement. Grinding the stone sludge increased the efficiency of the sludge by approximately 1% in the case of marble sludge, by 20.5% for sandstone sludge, and by 8.5% for mixed sludge. The results for compressive strength after 28 days indicated that 5% and 10% replacement with marble sludge and 5% replacement with mixed sludge appear to be promising. All three types of stone sludge in both variants (raw/ground), at substitution levels of 5 and 10%, showed a maximum reduction in compressive strength of up to 25% when compared with the reference mortar.
- With a 20% replacement of fine natural aggregate 0/4 mm fraction in the mortars, the reduction was within 10% in all cases after 28 days. At this replacement level, the mortars exhibited good workability and all the mechanical properties under investigation. Sandstone sludge achieved the best results in terms of mechanical properties. With a 20% replacement of fine aggregate, it even showed a slight improvement, and with the highest replacement rate of 50%, compressive strengths decreased by only approximately 20% compared with the reference.
- The results of strength tests on concrete containing waste stone sludge as a partial substitute for cement and fine natural aggregate show that it is possible to incorporate this sludge into concrete, provided that a certain reduction in strength is taken into account. A reduction of between 15–35% compared with the reference sample was observed, depending on the type of waste sludge used.
- Substituting cement and, above all, fine aggregate may offer economic benefits to concrete producers at this stage of the study. Sludge producers—stonemasonry workshops—offer sludge for free. This would result in savings on the additional costs associated with landfilling.

The results of the experimental tests carried out as part of the study demonstrated that the incorporation of waste stone sludge into concrete production at small-scale facilities specialising in the custom manufacture of non-standard concrete elements is possible to a limited extent. Furthermore, it is important to bear in mind that this approach to managing stone sludge may help to resolve landfill issues and steer this sector of the construction industry towards more sustainable development. However, several limitations and suggestions for future research need to be considered. The study was conducted on a laboratory scale, and the findings may show variations, primarily relating to the variability in the composition of individual stone sludge waste streams from different stonemasonry workshops.

## Figures and Tables

**Table 1:** Overview of the basic properties of Portland cement and stone sludge.

Properties	Unit	Portland Cement	Stone Sludge					
			Marble		Sandstone		Mixed	
			Raw	Ground	Raw	Ground	Raw	Ground
Specific gravity	kg/m^3^	3090	2750	2750	2700	2700	2730	2730
Specific surface (Blain)	m^2^/kg	406	316	551	44	117	340	533
Moisture	**%**	–	23.0	–	13.3	–	24.3	–

**Table 2:** Overview of the basic properties of superplasticizer.

Properties and Specifications	Superplasticizer
Density [kg/m^3^]	1.06
pH [kg/m^3^]	4.5
Dry mass content [–]	33.0
Maximum chloride content [% of weight]	0.1
Maximum alkaline content [% equivalent to Na_2_O]	1.0

**Table 3:** Composition of cement mortar formulations—substitution of raw cement with ground stone sludge (Stage I).

Mortar	Cement Substitute	Portland Cement	Stone Sludge	Standardised Sand	Water	Water/Cement Ratio
	%	g	g	g	g	–
Reference	0	900	–	2700	450	0.50
Marble	5	855	45	2700	450	0.53
	10	810	90	2700	450	0.56
	15	765	135	2700	450	0.59
Sandstone	5	855	45	2700	450	0.53
	10	810	90	2700	450	0.56
	15	765	135	2700	450	0.59
Mixed	5	855	45	2700	450	0.53
	10	810	90	2700	450	0.56
	15	765	135	2700	450	0.59

**Table 4:** Composition of cement mortar mixes—substitute of fine aggregate with unground stone sludge (Stage II).

Mortar	Fine Natural Aggregate 0/4 Substitute	Portland Cement	Stone Sludge	Fina Natural Aggregate 0/4	Water	Water Cement Ratio
	%	g	g	g	g	–
Reference	0	900	–	2700	450	0.50
Marble	20	900	540	2160	520	0.58
	35	900	945	1755	625	0.69
	50	900	1350	1350	740	0.82
Sandstone	20	900	540	2160	500	0.56
	35	900	945	1755	580	0.64
	50	900	1350	1350	640	0.71
Mixed	20	900	540	2160	520	0.58
	35	900	945	1755	640	0.71
	50	900	1350	1350	710	0.79

**Table 5:** Composition of concrete mix designs (Stage III).

Concrete	REF	REC 1			REC 2		
	–	Stone Sludge					
		Marble	Sandstone	Mixed	Marble	Sandstone	Mixed
	kg/m^3^						
Portland cement	389	350	350	350	350	350	350
Fine natural aggregate 0/4	870	696	696	696	566	566	566
Coarse aggregate 4/8	348	348	348	348	348	348	348
Coarse aggregate 8/16	522	522	522	522	522	522	522
Stone sludge	–	164	185	161	264	297	260
Water	154	187	163	183	205	193	203
Superplasticizer	3.8	2.8	2.8	2.8	2.8	2.8	2.8

**Table 6:** Test results for fresh mortar (Stage I).

Mortar	Cement Substitute	^1^ Stone Sludge—Raw		^1^ Stone Sludge—Ground	
		Consistency	Bulk Density	Consistency	Bulk Density
	%	mm	kg/m^3^	mm	kg/m^3^
Reference	0	185	2180	185	2180
Marble	5	180	2180	180	2150
	10	185	2200	190	2150
	15	180	2180	190	2180
Sandstone	5	180	2150	190	2120
	10	185	2160	190	2120
	15	180	2160	190	2160
Mixed	5	190	2180	190	2130
	10	190	2180	190	2150
	15	190	2210	190	2150

^1^ Dried sludge.

**Table 7:** Test results for fresh mortar (Stage II).

^1^ Mortar	Fine Natural Aggregate 0/4 Substitute	Consistency	Bulk Density
	%	mm	kg/m^3^
Reference	0	160	2180
Marble	20	160	2180
	35	160	2170
	50	170	2080
Sandstone	20	170	2210
	35	170	2180
	50	170	2020
Mixed	20	170	2250
	35	160	2080
	50	160	2080

^1^ Unground stone sludge only.

**Table 8:** Results for mortars in the hardened state at 7 days (Stage I).

Mortar	Cement Substitute	Stone Sludge—Raw			Stone Sludge—Ground		
		Bulk Density	Compressive Strength	Flexural Tensile Strength	Bulk Density	Compressive Strength	Flexural Tensile Strength
	%	kg/m^3^	MPa	MPa	kg/m^3^	MPa	MPa
Reference	0	2160	22.0	5.1	2160	22.0	5.1
Marble	5	2160	29.8	6.2	2120	29.7	6.2
	10	2160	30.3	6.4	2090	27.0	5.4
	15	2080	27.3	5.5	2110	24.5	6.1
Sandstone	5	1970	18.4	4.8	2140	28.6	6.2
	10	1980	15.1	4.2	2130	24.9	5.4
	15	1990	11.2	3.3	2120	21.4	5.5
Mixed	5	2070	27.7	5.7	2050	25.1	5.7
	10	2040	23.8	5.4	2060	23.0	5.4
	15	2120	23.2	4.9	2100	21.6	4.6

**Table 9:** Results for mortars in the hardened state at 28 days (Stage I).

Mortar	Cement Substitute	Stone Sludge—Raw			Stone Sludge—Ground		
		Bulk Density	Compressive Strength	Flexural Tensile Strength	Bulk Density	Compressive Strength	Flexural Tensile Strength
	%	kg/m^3^	MPa	MPa	kg/m^3^	MPa	MPa
Reference	0	2150	38.2	6.0	2150	38.2	6.0
Marble	5	2140	40.0	7.5	2130	40.6	7.5
	10	2150	37.1	6.7	2100	36.7	6.9
	15	2090	30.1	6.4	2080	30.5	6.2
Sandstone	5	2100	31.5	5.5	2110	33.4	6.2
	10	2140	28.6	6.1	2140	30.9	6.4
	15	2030	21.5	4.0	2110	29.2	6.0
Mixed	5	2080	34.6	6.3	2120	35.0	6.5
	10	2060	28.9	5.9	2080	30.7	6.3
	15	2030	27.3	5.6	2090	30.6	5.9

**Table 10:** Results for mortars in the hardened state at 7 and 28 days (Stage II).

Mortar	Fine Natural Aggregate 0/4 Substitute	Bulk Density	Compressive Strength	Flexural Tensile Strength	Bulk Density	Compressive Strength	Flexural Tensile Strength
	%	kg/m^3^	MPa	MPa	kg/m^3^	MPa	MPa
		7 Days			28 Days		
Reference	0	2160	30.3	5.8	2140	33.6	7.5
Marble	20	2130	28.1	5.1	2110	32.8	6.9
	35	2080	25.2	5.0	2020	31.1	5.4
	50	2020	19.9	4.5	1990	25.8	5.7
Sandstone	20	2150	27.7	5.8	2110	36.0	7.1
	35	2070	23.0	5.0	2070	31.5	6.5
	50	2040	19.5	4.5	2000	26.4	5.6
Mixed	20	2130	24.5	5.7	2040	28.7	6.7
	35	2040	20.8	4.9	2020	25.9	6.5
	50	1950	15.4	3.8	1910	21.3	4.5

**Table 11:** Results for concrete in the hardened state at 7 and 28 days (Stage III).

Concrete		Bulk Density	Compressive Strength	Bulk Density	Compressive Strength	Flexural Tensile Strength
		kg/m^3^	MPa	kg/m^3^	MPa	MPa
		7 Days		28 Days		
Reference	–	2320	57.6	2310	68.2	6.8
REC 1	Marble	2280	45.4	2250	56.7	5.7
	Sandstone	2270	44.8	2250	54.0	6.3
	Mixed	2230	35.8	2190	46.4	5.0
REC 2	Marble	2300	42.9	2240	51.9	5.8
	Sandstone	2260	39.5	2180	48.6	5.5
	Mixed	2270	38.0	2180	53.0	5.2

**Table 12:** Results of the determination of the depth of penetration of water under pressure.

Concrete	Type of Stone Sludge	Depth of Penetration of Water Under Pressure
	–	mm
Reference	–	9
REC 1	Marble	11
	Sandstone	10
	Mixed	19
REC 2	Marble	18
	Sandstone	20
	Mixed	23

## Data Availability

The original contributions presented in this study are included in the article. Further inquiries can be directed to the corresponding author.
